# Fertility preservation for pediatric patients with hemoglobinopathies: Multidisciplinary counseling needed to optimize outcomes

**DOI:** 10.3389/fendo.2022.985525

**Published:** 2022-10-24

**Authors:** Bronwyn S. Bedrick, Taylor P. Kohn, Lydia H. Pecker, Mindy S. Christianson

**Affiliations:** ^1^Department of Gynecology and Obstetrics, Johns Hopkins University School of Medicine, Baltimore, MD, United States; ^2^Department of Urology, Johns Hopkins University School of Medicine, Baltimore, MD, United States; ^3^Department of Medicine, Division of Adult Hematology, Johns Hopkins University School of Medicine, Baltimore, MD, United States; ^4^Department of Gynecology and Obstetrics, Division of Reproductive Endocrinology and Infertility, Johns Hopkins University School of Medicine, Baltimore, MD, United States,

**Keywords:** fertility preservation, sickle cell disease, beta thalassemia, oocyte cryopreservation, ovarian tissue cryopreservation, sperm cryopreservation

## Abstract

Hemoglobinopathies are autosomal recessive disorders that occur when genetic mutations negatively impact the function of hemoglobin. Common hemoglobinopathies that are clinically significant include sickle cell disease, alpha thalassemia, and beta thalassemia. Advancements in disease-modifying and curative treatments for the common hemoglobinopathies over the past thirty years have led to improvements in patient quality of life and longevity for those who are affected. However, the diseases, their treatments and cures pose infertility risks, making fertility preservation counseling and treatment an important part of the contemporary comprehensive patient care. Sickle cell disease negatively impacts both male and female infertility, primarily by testicular failure and decreased ovarian reserve, respectively. Fertility in both males and females with beta thalassemia major are negatively impacted by iron deposition due to chronic blood transfusions. Hematopoietic stem cell transplant (HSCT) is currently the only curative treatment for SCD and transfusion dependent beta thalassemia. Many of the conditioning regimens for HSCT contain chemotherapeutic agents with known gonadotoxicity and whole-body radiation. Although most clinical studies on toxicity and impact of HSCT on long-term health do not evaluate fertility, gonadal failure is common. Male fertility preservation modalities that exist prior to gonadotoxic treatment include sperm banking for pubertal males and testicular cryopreservation for pre-pubertal boys. For female patients, fertility preservation options include oocyte cryopreservation and ovarian tissue cryopreservation. Oocyte cryopreservation requires controlled ovarian hyperstimulation (COH) with ten to fourteen days of intensive monitoring and medication administration. This is feasible once the patient has undergone menarche. Follicular growth is monitored *via* transvaginal or transabdominal ultrasound, and hormone levels are monitored through frequent blood work. Oocytes are then harvested *via* a minimally invasive approach under anesthesia. Complications of COH are more common in patients with hemoglobinopathies. Ovarian hyperstimulation syndrome creates a greater risk to patients with underlying vascular, pulmonary, and renal injury, as they may be less able to tolerate fluids shifts. Thus, it is critical to monitor patients undergoing COH closely with close collaboration between the hematology team and the reproductive endocrinology team. Counseling patients and families about future fertility must take into consideration the patient’s disease, treatment history, and planned treatment, acknowledging current knowledge gaps.

## Introduction

Hemoglobin is an oxygen-carrying protein comprised of four subunits: two alpha chains and two non-alpha globin chains. In a healthy adult, approximately 95-98% of hemoglobin is in the form of HbA1, which consists of two alpha and two beta chains; the remaining small percentage of hemoglobin is in the form of HbA2 (two alpha and two delta chains) and HbF (two alpha and two gamma chains) (1). Almost 2,000 hemoglobin gene variants have been described (2). However, most variants are not associated with clinically significant disease. Indeed, it is estimated that 24% of the world population carry at least one altered globin gene, but only 5% carry a clinically significant variant (3). Hemoglobinopathies are autosomal recessive disorders: sickle cell disease, alpha thalassemia, and beta thalassemia.

Advancements in disease-modifying and curative treatments for the common hemoglobinopathies over the past thirty years have led to improvements in patient quality of life and longevity for those who are affected. However, the diseases, their treatments and cures may pose infertility risks. Expanding opportunities to preserve fertility in childhood are thus relevant for children with these common genetic conditions.

In this article, we discuss the indications and complications of fertility preservation in pediatric patients with hemoglobinopathies, specifically sickle cell disease and beta thalassemia. We also review their etiologies and impact on fertility and summarize their main disease modifying treatment options, focusing on the use of hydroxyurea and hematopoietic stem cell transplant. Finally, we review healthcare and research disparities in this field.

## Overview of common hemoglobinopathies

### Sickle cell disease

Sickle cell disease (SCD) refers to a group of hemoglobinopathies characterized by two β-globin gene mutations or deletions, at least one of which is the point mutation that leads to the production of hemoglobin S (HbS) (**Table 1**). An adenine-to-thymine substitution in the sixth codon of the beta-globin chain results in HbS. This substitution creates an insoluble polymer that distorts the cellular membrane and promotes the characteristic red blood cell sickling in deoxygenated states. The inability of HbS to deform normally results in hemolysis, a shortened red blood cell lifespan, and a hypercoagulable state. Additionally, the sickled red blood cells may become entrapped within vessels, leading to vascular occlusion and ischemia that promotes further sickling. This is the mechanism responsible for vaso-occlusive pain crises (VOC), acute chest syndrome (ACS), stroke, splenic sequestration, neuropathy, osteonecrosis, and recurrent infections, among other severe complications of SCD (4).

**Table 1:** Hemoglobinopathy pathophysiology, major treatment modalities, and impact on fertility.

Hemoglobinopathy	Pathophysiology	Fertility Effects	Treatments	Treatment Fertility Effect
Beta Thalassemia	Alpha globin chain precipitates due to globin chain imbalance, leading to hemolysis and ineffective hematopoiesis	Fertility effects thought to be secondary to chronic transfusions	Blood transfusions	- Iron overload leads to hypothalamic hypogonadism, impaired leptin synthesis, delayed puberty - Diminished sperm production
			HSCT	Chemotherapeutic agents and radiation may lead to diminished ovarian reserve, diminished sperm production, varying degree of gonadal failure and infertility
Sickle Cell Disease	Hemoglobin becomes insoluble polymer, distorts the cellular membrane and promotes red blood cell sickling in deoxygenated states; results in hemolysis, entrapment, and hypercoagulability	Male: - hypogonadism - impaired spermatogenesis - delay in sexual maturation - Erectile dysfunction Female - delay in sexual maturation - diminished ovarian reserve	Pain management: NSAIDs, opioids	Opioids: inhibits GnRH NSAIDs: impairs ovulation, fertilization, and implantation
			Blood/exchange transfusions	- Iron overload leads to hypothalamic hypogonadism, delayed puberty
			Hydroxyurea	- Diminished sperm production and concentration - Diminished ovarian reserve - In pregnancy: birth defects, FGR
			HSCT	Chemotherapeutic agents and radiation may lead to diminished ovarian reserve, diminished sperm production, varying degree of gonadal failure and infertility

HSCT, hematopoietic stem cell transplant; GnRH, gonadotropin releasing hormone; NSAIDs, nonsteroidal anti-inflammatory drugs; FGR, fetal growth restriction.

SCD occurs when an individual is homozygous for HbS (i.e., HbSS, sickle cell anemia) or compound heterozygous with another beta globin gene mutation.

### Beta thalassemia

Thalassemia arises from globin chain imbalance due to mutations in one of the four alpha subunit genes or one of the two beta subunit genes. An imbalance in the production of alpha and beta globin chains produces unpaired globin chains that precipitate within red blood cells, resulting in hemolysis and ineffective hematopoiesis. Thalassemia severity depends on the type of genetic defect (i.e., missense versus full deletion) and on the number of genes affected.

Beta thalassemia is caused by mutations in the beta globin gene. Some mutations reduce expression of the beta subunit (β+), whereas others result in complete loss of expression from that allele (β^0^). Individuals with one functional beta globin (β/β+ or β/β^0^; beta thalassemia minor) are asymptomatic carriers. Patients with some normal beta globin production (β+/β+ or β+/β^0^; beta thalassemia intermedia) usually have mild to moderate anemia, although patients may require chronic transfusions. Beta thalassemia major (BTM) is characterized by severe anemia that results when both beta globin genes have deletions (β^0^/β^0^) or when a deletion is paired with another mutation that severely decreases beta globin expression (5).

When patients are dependent on transfusions for survival, regardless of genotype, they are said to have transfusion-dependent thalassemia (6). These patients usually have BTM and without treatment or cure, they are at risk of growth impairment, skeletal abnormalities, hepatosplenomegaly, and death within the first two decades of life (5). Life expectancy for individuals with BTM has significantly (7) improved over the years. In the 1970s, half of patients died before the age of 12 (8); however, many patients are now living into their 50s or 60s, making normal puberty important and parenthood viable (9).

Given the prevalence and severity of BTM and SCD, this review article will focus on these hemoglobinopathies. We note that a small, but growing number of people with alpha thalassemia major are surviving. These patients, like those with beta thalassemia major are at risk for iron overload and gonadotoxicity during HSCT. Given the lack of data, however, we do not focus on these patients (7, 10).

## Impact of hemoglobinopathies on fertility

### Sickle cell disease

#### Male infertility risks

Studies suggest that males with SCD are at risk for infertility as a result of both hypergonadatropic hypogonadism from vaso-occlusive induced testicular ischemia as well as hypogonadotropic hypogonadism from chronic transfusion-induced iron deposition in the hypothalamus and pituitary (11–13). Indeed, studies have demonstrated that approximately 24% of adult men with SCD are hypogonadal from both hypergonadatropic hypogonadism as well as hypogonadotropic hypogonadism (13–16). As a result, adolescent males may experience a delay in sexual maturation by one to two years, and boys with more severe genotypes (HbSS, HbSβ^0^) experiencing greater delays (13, 17).

Infertility in males with SCD can also occur as a result of severe erectile dysfunction. Vaso-occlusion of the corpus cavernosum can result in priapism and repeated vasoocclusive episodes with priapism can result in high rates of erectile dysfunction, with some studies demonstrating as many as 48% of men will have impaired erectile function at an average age of 28 years old (18). Priapism is a true SCD emergency as the risk of erectile dysfunction increases with prolonged episodes of priapism (19, 20). Severe cases of erectile dysfunction can make spontaneous reproduction difficult and limit future fertility and may even require penile prosthesis to achieve an erection necessary for intercourse.

Even in eugonadal men with SCD, spermatogenesis is often affected; impaired semen parameters have been observed in men with normal FSH, LH and testosterone levels, possibly due to testicular infarction (21, 22). In one study, 91% of patients with SCD and taking no disease modifying therapy had an abnormality on semen analysis. The most common abnormality being impaired motility, though total motile counts were still on average around 32 million motile sperm (23). Despite a high published rates of abnormalities on semen analysis in men with sickle cell disease, in a large retrospective registry study of patients with sickle cell disease, Gordeuk et al. found that among 1018 men with sickle cell disease, 620 pregnancies conceptions had been reported for a rate of 0.61 per man (24).

Men with SCD can conceive an unassisted pregnancy with a partner, though no largescale study has assessed the frequency of infertility in this population, and further studies are needed to determine.

#### Female infertility risks

The majority of data on female sexual development in SCD is from the 1960s-1990s. These studies demonstrated that females with SCD achieve sexual development and undergo menarche at later ages than unaffected females (25), with more severe genotypes (HbSS, HbSβ^0^) having greater delays than those with less severe hemoglobinopathies (HbSβ^+^ or HbSC) (13, 26). The delay in menarche is thought to be constitutional (13, 26), and age of menarche is consistent with bone age (27). Once menarche is reached, however, patients can be expected to have regular menstruation (26). The effects of disease-modifying therapy on age of menarche remains poorly defined (28).

The extent to which fertility is impacted in female patients with SCD is also unclear. Historically, lower pregnancy rates among women with SCD was used as a surrogate for fertility (29), but this approach to estimating fertility is limited. Women with SCD have multiple risks for reduced ovarian reserve: chronic inflammation, oxidative stress, and ovarian ischemia and reperfusion injuries (30, 31). Three studies have demonstrated normal anti-müllerian hormone (AMH) levels in untreated adolescent and young adults with SCD (32–34). However, women with SCD experience a more rapid decline in ovarian reserve, with lower levels of AMH than age matched controls (33, 35, 36). Females with SCD develop diminished ovarian reserve (DOR) at younger ages (25-30 years) than age-matched women (33, 34). Yet, no studies to date define the definitive risk of infertility in this population (28). Interestingly, in 2021 Mamsen et al. evaluated ovarian health markers in adolescent females with hemoglobinopathies. They found no difference in ovarian follicular density, morphology, and expression of follicular and oocyte proteins between those with SCD and health age-matched controls, suggesting that the primordial follicle pool is normal in this population (37).

### Beta thalassemia

Delayed sexual development, menarche, and hypothalamic hypogonadism are common in adolescents with BTM (38) but are these thought to be secondary to iron deposition from chronic transfusions rather than a consequence of the disease itself (39). Impact of chronic transfusion on pubertal development and fertility and prevention options will be further discussed in the next section.

For both SCD and BTM, a patient’s disease and disease severity, may impact which fertility preservation treatment options are available to them and the success of their fertility treatment. When counseling patients and guardians about treatment options, it is important to consider how the individual’s unique disease presentation may impact success.

## Palliative and disease modifying therapies as potential infertility risks

### Pain management

Chronic pain and opioid use are common sequelae of SCD. Additionally, almost 70% of patients with BTM report recent pain (40). Opioids have been shown to suppress the hypothalamic-pituitary-gonadal axis through inhibiting gonadotropin releasing hormone (41). Indeed, women taking opioids chronically have an approximately 50% reduction in estradiol and testosterone levels, a 30% reduction in gonadotropins (42), and may experience menstrual cycle disruption (42). Chronic opioid use also reduces testosterone levels in males (43) and leads to lower sperm motility and morphology (44) as well as increased DNA fragmentation (43).

The use of nonsteroidal anti-inflammatory drugs (NSAIDs) can impact fertility through inhibition of cyclooxygenase 2 (COX-2), leading to reduced prostaglandin synthesis and impairments in ovulation, fertilization, and implantation (45, 46). While small studies have linked impaired ovulation and infertility with NSAID use, the impact of chronic NSAID use on ovarian reserve and fertility is not well understood (30). Furthermore, polypharmacy may obfuscate associations between analgesic medications and fertility in patients with SCD or BTM.

### Blood transfusion

Red blood cell transfusion or exchange is a cornerstone for symptomatic management and prevention in patients with SCD and BTM. For patients with SCD, these transfusions/exchanges dilute sickle cell hemoglobin and thus reduce the sequalae of sickling. For patients with BTM, transfusions supply normal red blood cells and inhibit ineffective erythropoiesis (47). These patients often undergo transfusions every three to four weeks (5).

Despite clear benefits, chronic transfusions often lead to iron overload, which has both direct and indirect impact on gonadal function. Interestingly, iron deposition appears to be a greater issue for individuals with BTM than those with SCD (48, 49), and effects from iron deposition are the major complications associated with BTM. For example, in a study of 73 patients with BTM and SCD who received chronic transfusions, 33% of BTM had gonadal failure compared to 0% of SCD patients (48). A similar 2006 study found that 40% of patients with BTM had hypogonadism, and they were eight times as likely to have hypogonadism as patients with SCD (49). Chelation therapy thus becomes essential for those receiving chronic blood transfusions and should often be initiated prior to puberty in order to encourage normal development (50). However, progressive deposition in the hypothalamus and pituitary will occur even in the setting of chelation therapy (51).

Hypothalamic hypogonadism may result from direct iron deposition in the hypothalamus, pituitary, and reproductive organs as well as free radial oxidative stress (39, 52–54). The anterior pituitary is particularly sensitive to iron deposition and demonstrates evidence of iron accumulation within the first decade of life. Damage to the anterior pituitary leads to disturbances in gonadotropic hormones and may lead to pubertal delays or arrest. In fact, hypothalamic hypogonadism is the most common endocrinopathy affecting individuals with BTM (38). It is estimated that 70% of boys with beta thalassemia intermedia or major will develop hypogonadotropic hypogonadism (55), and over 50% of females will not reach menarche spontaneously (38). In addition to iron deposition in the hypothalamus and pituitary, gonadal iron deposition may occur (56). However, ovarian function appears to be preserved as evidenced by an age-appropriate ovarian response to hyperstimulation (57, 58). Furthermore, ovarian tissue preserved for fertility preservation in females with BTM demonstrate normal follicular density and morphology (37).

Iron deposition also negatively impacts fertility in patients with BTM include impaired leptin synthesis and disruption of liver and pancreatic function, which are involved in hormone and antioxidant metabolism (39). It has been suggested that iron deposition in adipose tissue disrupts the production of leptin, a hormone now believed to be vital for the pubertal development. In a study of 101 adolescents with BTM, Perrone et al. found significantly lower leptin levels than expected for Tanner stage 1-4 males and stage 3-5 females (59). In a separate study, Dedoussis et al. found that leptin serum levels were significantly lower for BTM patients who received either sporadic or chronic transfusions than normal and that leptin level was negatively correlated with levels of transferrin reception for those who were transfusion dependent (60).

Males with transfusion dependent beta-thalassemia have high rates of oligospermia and azoospermia, but conception is still possible. In a study of 52 men, 60% were normospermic, 17% were oligospermic, and 23% were azoospermic (61). For men with impaired spermatogenesis, spermatogenesis can be induced with exogenous gonadotropin stimulation, with human chorionic gonadotropin (hCG) alone or combined with human menopausal gonadotropin, thus making paternity possible (9, 62–64) Indeed, in a survey of ten thalassemia centers, including 738 transfusion-dependent men over the age of 18, 75% of those married or living with a partner conceived a pregnancy within the first two years of the marriage. Of these pregnancies, 79% occurred *via* natural conception and 15% of men required exogenous gonadotropin stimulation (65).

### Hydroxyurea

Approved by the FDA in 1998 for use in adults with sickle cell anemia, hydroxyurea has dramatically improved patient quality of life and reduced disease complications. Patients taking hydroxyurea are less likely to be hospitalized or require transfusions. Studies have also found improvements in long-term survival and reduced risk of stroke (66–71).

Hydroxyurea inhibits ribonucleotide reductase and thus cell cycle specific DNA replication. Through unclear mechanisms, hydroxyurea shifts expression of the beta globin locus resulting in increased production of HbF and decreased production of HbS. As a result of decreased HbS concentration, hemoglobin is less prone to polymerization and sickling. Hydroxyurea also decreases circulating leukocytes and reticulocytes, increases red blood cell volume, and improves cellular deformability, thereby reducing painful events (72).

Given substantive improvements in patient symptoms and markers of disease control, the National Heart, Lung, and Blood Institute (NHLBI) recommends offering hydroxyurea in pediatric patients over 9 months of age, regardless of clinical severity (68). However, the optimal time to start hydroxyurea therapy has not been established, and other national guidelines recommend starting at later ages (73). Additionally, in patients not taking hydroxyurea, it may be recommended prior to bone marrow transplant to reduce the risk of rejection and improve chance of engraftment (74). While there have been some studies suggesting benefit in the use of hydroxyurea in patients with BTM (75), these results are not widespread and its use in this population is uncommon (5).

Hydroxyurea use may be lifelong for patients with SCD. While there is strong evidence as to the myelosuppressive effects of hydroxyurea, data on other long-term effects, such as on infertility, are conflicting. The National Toxicology Program (NTP) Center for the Evaluation of Risks to Human Reproduction (CERHR) gave hydroxyurea use in pregnancy its second highest concern level due to risk for birth defects and intrauterine growth restriction (76). CERHR also ranks use of hydroxyurea in post-pubertal males as “highly concerning.”

For men, hydroxyurea owes its rank of “highly concerning” to its impact on sperm parameters. In men, treatment with hydroxyurea has been shown to significantly impair total sperm concentration (pretreatment: 38.5 million sperm/mL; post treatment: 18.46 million sperm/mL) but forward motility remains similar (pretreatment: 28.6%; post treatment: 29.4%) (23). In another study of men with SCD on hydroxyurea therapy, 20% developed oligospermia and 10% developed azoospermia (77). The impact on spermatogenesis extends to prepubertal males as well. In one study, two young males initiated on hydroxyurea at ages 10 and 16 were found to have severe oligospermia eight years after treatment initiation; two others who began hydroxyurea at ages 8 and 11 were found to be azoospermic 15 and 12 years later, respectively (78).

Despite hydroxyurea’s deleterious impact on semen parameters, studies have shown normalization of semen parameters after discontinuing the medication. In one study, almost 75% of men who stopped hydroxyurea for three months had normalization of their semen parameters (77). In a recent study by Joseph et al., there was no difference in semen volume, sperm concentration, total sperm count, or spermatozoa motility, morphology, and vitality between men who received hydroxyurea prior to puberty and men who were hydroxyurea naïve. In this study, men who had a history of hydroxyurea use stopped hydroxyurea on average two and a half years prior to semen analysis (79). These studies suggest that while hydroxyurea may have more severe effects when started in the prepubertal period, the effects are potentially reversible. However, the reliability and duration to recovery of spermatogenesis after hydroxyurea has not been well elucidated, and so sperm banking or testicular tissue banking may be considered prior to initiating hydroxyurea therapy.

While hydroxyurea is considered ‘low risk’ for infertility in women, it is associated with diminished ovarian reserve in three small studies of people with sickle cell anemia (hemoglobin SS and hemoglobin S beta-null thalassemia). During the decade-long follow up of the Multicenter Study of Hydroxyurea (MSH) randomized control trial, AMH was lower for women who were on hydroxyurea than for those not taking it (33). In 2015, Elchuri et al. compared 10-21 year-old females with SCD who received supportive care, hydroxyurea, or underwent bone marrow transplantation. They found that 24% of patients treated with hydroxyurea had diminished ovarian reserve as defined by AMH <5% of expected for age-matched controls (32). Moreover, among patients taking hydroxyurea, those with DOR had been taking the medication for 2.8 years longer on average than those without DOR. No patient receiving hydroxyurea in this study met criteria for premature ovarian insufficiency as defined as FSH is > 40 IU/L (32). In another single center analysis of ovarian reserve in 26 women with sickle cell anemia, all (n=5) women diagnosed with DOR were taking hydroxyurea (34). Whether markers of ovarian reserve normalize when hydroxyurea is stopped and whether hydroxyurea impairs fertility, ovarian hyperstimulation, or oocyte quality is not established. Given the potential to affect oocyte quantity and quality, the potential risks associated with its use must be weighed against the potential benefit of reduced rejection and graft versus host disease risk.

### Hematopoietic stem cell transplant

Hematopoietic stem cell transplant (HSCT) is currently the only curative treatment for SCD and transfusion dependent beta thalassemia. It is recommended when symptoms are no longer controlled with supportive care and medical management or for those who with serious disease complications. For patients with SCD who underwent HLA-matched transplants, greater than 90% sustained engraftment and resolution of painful crises (80). Similar improvements are seen in patients with BTM. To date, more than 5,000 of HSCT have been performed for these disorders (81–83).

For patients with SCD, HLA matched sibling donors result in the highest event-free survival. However due to limited availability of HLA-matched siblings, haploidentical, matched unrelated, and mismatched unrelated donation are possible and mostly offered on an experimental basis (84). Age at transplantation impacts morbidity and mortality, which is an important consideration for fertility preservation. When HSCT is performed prior to 16 years of age, the 5-year survival is 95% compared to 81% at older ages (82), and children <10 years old have decreased mortality compared to those transplanted 10-21 years old (85). Similarly, for BTM HLA identical transplantation at younger ages (<14 years) improves outcomes and survival (83, 86) due to higher rates of disease complications with age; transplant related mortality is <5% if performed prior to 5 years old (83).

A variety of condition regimens for HSCT are described. The first successful regimens were myeloablative. However, given the intolerability of these regimens for those with severe disease, nonmyeloablative conditioning regimens have been developed. Many of the conditioning regimens contain chemotherapeutic agents with known gonadotoxicity, such as cyclophosphamide. Whole body radiation may also be included. The commonly used radiation dose (2-3Gy) is less than the effective sterilizing dose (>14 Gy in females; > 6 Gy in males) but falls within ranges known to cause significant gonadal damage (87–89). Despite the potential for significant deleterious gonadal damage, most clinical studies on toxicity and impact of HSCT on long-term health do not evaluate gonadal function or fertility. From the studies that do (**Table 2**), it is evident that gonadal failure is common, but the impact is variable and unpredictable (115). Additionally, younger age at transplant and male sex appear to reduces risk of gonadal failure (109).

**Table 2:** Clinical studies of hematopoietic stem cell transplant in patients with sickle cell disease and beta thalassemia that evaluated reproductive function.

Authors, year	Diagnosis	Sample Size	Median age at time of HSCT(range)	Conditioning regimen	Follow up time	Impact on reproductive function
De Santis et al., 1991 (90)	BTM	N=30	Mean 12.9 years (9.3-17.2 years)	Busulfan/cyclophosphamide	0.7-5.1 years	- 12/15 females had elevated levels of LH and FSH - 15/15 males had normal LH, FSH, testosterone
Vermylen et al., 1998 (91)	SCD	N=50	7.5 years (0.9-23 years)	Busulfan/Cyclophosphamide +/- total lymphoid irradiation or ATG	0.3-11 years	- 2/2 postpubertal females developed secondary amenorrhea - 5/6 prepubertal females had primary amenorrhea and required hormone replacement - 1/6 prepubertal female had spontaneous menarche - 6/6 males adolescent boys had normal sexual development - 4/6 males had decreased testosterone and elevated FSH -1/6 males had elevated LH
Slavin et al., 1998 (92)	Malignant and non-malignant hematologic diseases	N=26 (1 with BTM)	31 years (1-61 years)	Fludarabine/ATG/low-dose busulfan	Median: 8 months	1 19-year-old female regained menstruation
Bernaudin et al., 2007 (93)	SCD	N=87	9.5 years (2-22 years)	Busulfan/cyclophosphamide/ ATG	2-17.9 years	- 7 postpubertal females developed amenorrhea, low estradiol, elevated FHS and LH levels - “most” of prepubertal females required hormone therapy - 2 prepubertal girls underwent spontaneous puberty - All males had normal testosterone, FSH, and LH levels
Brachet et al., 2007 (94)	SCD	N=30	7.2 years (2.3-14.2 years)	Busulfan/cyclophosphamide	2.5-17.3 years	- 7/10 females had amenorrhea - 1 spontaneous pregnancy/live birth - 9/9 males underwent spontaneous puberty 9/9 males had normal/low-normal testosterone -1/2 males had azoospermia
Lukusa et al., 2009 (95)	SCD	N=10	32 years (10-34 years)	Busulfan/cyclophosphamide +/- total lymphoid irradiation	8-21 years	- 5/5 spontaneous puberty - 3/6 azoospermia - no pregnancies fathered
Hsieh et al., 2009 (96) (supplemental material)	SCD	N=10	26 years (16-45 years)	Total-body irradiation (3Gy)/alemtuzumab	15-54 mo	1.25 to 4.5 years post HSCT: - Range of FSH 5.8-179 units/L; LH 2-98.4 units/L - 1 female patient had FSH >40 units/L 0.5 years after HSCT; < 40 m/L FSH 1 and 2 years after - 1 pregnancy/ delivery - 1 female has regular menses on oral contraception - Range of total testosterone 191-1230 ng/dL; free testosterone 4.1-40.7 ng/dL - 1 male on testosterone replacement
Walters et al., 2010 (97)	SCD	N=55	<16	Busulfan/cyclophosphamide	3-12.4 years	- 9/12 females had amenorrhea - 2 spontaneous pregnancies/live births - 8/11 males had low testosterone
Majumdar et al., 2010 (98)	SCD	N=10	10.1 years (2.8-16.3 years)	Busulfan/cyclophosphamide/ thyroglobulin	2.9-11 years	-2/7 had FSH >40 mIU/mL more than 3 years post-transplant -2/3 females >14 have POF
Dallas et al., 2013 (99)	SCD	N=22	11.1 years (5.4-17.4 years)	Busulfan/cyclophosphamide/ thyroglobulin	0.9-12.3 years	-5/9 males had normal gonadal function (normal LH, FSH, testosterone) - 3/9 males had hypogonadism with normal testosterone -1/9 male required testosterone - 2/4 females developed POF requiring therapy -2/4 females had normal cycles and hormone levels - 1 pregnancy (unspecified if spontaneous)
Hsieh et al., 2014 (100)	SCD	N=30	Not reported (16-65 years)	Alemtuzumab/total-body irradiation (300 cGy)/sirolimus	1-8.6 years	- spontaneous conception for 2 women and 2 men after transplant (no specifics)
Bhatia et al., 2014 (101)	SCD	N=17	8.9 years (2.3-20.2 years)	Busulfan/fludarabine/ alemtuzumab	135–2731 days	- Semen analyses, testosterone, LH, FSH levels measured in postpubteral males - AMH, estradiol, LH, FSH levels measured in postpubertal females No results reported
Dedeken et al., 2014 (102)	SCD	N=50	8.3 years (1.7-15.3 years)	Busulfan/cyclophosphamide; Busulfan/cyclophosphamide/ ATG	0.4-21.3 years	- 3/12 spontaneous puberty - 1 female had 2 spontaneous pregnancies - 1 female had POF which recovered spontaneously and had normal pregnancy
Soni et al., 2014 (103)	SCD	N=15	5 years (1.5-18 years)	Busulfan/cyclophosphamide/ thyroglobulin	0.9-7.5 years	-2/3 females had gonadal dysfunction
Maheshwari et al., 2014 (104)	SCD	N=16	6.2 years (1.2-19.3 years)	Busulfan/cyclophosphamide/ thyroglobulin	1.3-9 years	-2/5 (1 male and 1 female) had gonadal dysfunction requiring hormone replacement
Elchuri et al., 2015 (32)	SCD	N=56	10-21 years	Supportive care vs hydroxyurea (≥20mg/kg for ≥12mo vs HSCT (busulfan and cyclophosphamide)	n/a	- Mean AMH was 17.1 (supportive care), 13.4 (HU), and <0.57pmol/L (HSCT) - POI was found in 0% (Supportive care and HU) and 89% (HSCT) patients
King et al., 2015 (105)	SCD, BT	N=52	11.5 years (0.8-20.3 years)	Reduced intensity conditioning: alemtuzumab/ Fludarabine/melphalan	0.75-11.8 years	- Resumption of menses within a year of transplant in 4 teenagers - “maintained fertility” (not defined) in 3 females
Madden et al., 2016 (106)	Mixture of diagnoses (immune, metabolic, hemoglobinopathy) Results not differentiated by diagnosis	N= 43 (10 had hemo-globinopathy)	3.4 years (1.5 mo-20 years)	Reduced intensity conditioning with Alemtuzumab/Fludarabine/mephalan	2 to 8 years	- 1 of 17 had hypogonadism (also had chronic GvHD) - 3 of 3 postpubertal girls resumed menstruation; 2 had normal pregnancies - 9 of 11 age-appropriate Tanner development
Marzollo et al., 2017 (107)	SCD	N=11	6.5 years (4-16.3 years)	Treosulfan/fludarabine/ATG/ thiotepa	0.8-6.5years	- 3/4 had normal pubertal development -1/4 had secondary hypogonadism
Santarone et al., 2017 (108)	BTM	Males: N=8 Females: N=15	Males: 15 years (4-24 years) Females: 14 years (2-21 years)	Busulfan (total dose, 14 mg/kg) and cyclophosphamide (total dose, 200 mg/kg) as conditioning therapy and cyclosporine and short-course methotrexate as GvHD prophylaxis	Median 24 years (10-33 years)	-15 women achieved 27 pregnancies, 21 were achieved *via* natural conception, 6 *via* IVF - 2 miscarriage - 3 abortions (2 intended, 1 unintentional) - 22 live births - 8 men achieved 15 pregnancies with their partner, all *via* natural conception - 1 intended abortion - 14 live births
Rahal et al., 2018 (109)	BT	N=99	5.9 years (8mo-26 years)	Busulfan/cyclophosphamide; busulfan/fludarabine +/- thiotepa 3 other regimens including radiation	2-30 years	- Hypogonadism present in 56% of females and 14% of males - 6/6 females had secondary amenorrhea; 5 had hypogonadism -12/33 females had spontaneous and normal puberty -21/33 females had delayed puberty - 11/27 females had 1+ successful pregnancy, 2 required oocyte donation (both had delayed puberty) -4/22 males had delayed puberty; 3 developed hypogonadism - 18/22 males had normal pubertal development -4/21 males had fathered 1+ children (1 required IVF for hypogonadism and oligo-asthenozoospermia)
Zhao et al., 2019 (110)	SCD	N=3	14 years (11-15 years)	Alemtuzumab/fludarabine/ melphalan	>1 year post transplant	-3/3 normal testosterone - 2/3 azoospermia - no pregnancies fathered
Elchuri et al., 2020 (111)	SCD	N=40	9 years (6-34 years)	Busulfan/cyclophosphamide	1.1-18.5 years	- 21/21 females had DOR; 18 had undetectable AMH - 10/21 females had POI - 1 female had a spontaneous pregnancy/livebirth - 16/16 males had normal testosterone - no males fathered pregnancies
Bernaudin et al., 2020 (112)	SCD	N= 234	8.4 years (2.2-28.9 years)	Busulfan, cyclophosphamide at 200 mg/kg and rabbit ATG at different doses	0.1-27.6 years	- 14/14 postpubertal females were amenorrheic within 1 year and required hormone replacement - “Most” of 32 pre-pubertal females required hormone therapy - 9 of 32 prepubertal girls underwent spontaneous puberty - 6 spontaneous pregnancies - 2 females had orthotopic ovarian fragment autograft; both recovered ovarian function; 1 conceived twice - All males who were of pubertal age had normal development, normal testosterone, FSH, LH - 3 males had fathered spontaneously
Rostami et al., 2020 (61)	BTM	N=43 (HSCT) N=52 (chronic transfusion)	Range 16-41 years	Cyclophosphamide/busulfan		- 33% of entire cohort had hypogonadism HSCT cohort -26% had dry ejaculate - 51% had azoospermia - 12% had oligospermia Transfusion cohort - 10% had dry ejaculate - 23% had azoospermia -17% had oligospermia
Alzahrani et al., 2021 (113)	SCD	N=122	29 years (10-65 years)	Alemtuzumab/total body irradiation (3 Gy)	0.6-14.9 years	- 7 females had 1+ spontaneous pregnancies - 7 males fathered 1+ pregnancies spontaneously
Boga et al., 2022 (114)	SCD	N= 49	Not reported (18-45 years)	Busulfan/cyclophosphamide/ Fludarabine/total body irradiation (2Gy)	>2 years after transplant	- 15/22 females had documented amenorrhea - 7/22 females without amenorrhea were on hormonal support - All women had AMH <1ng/mL 2 years after transplant - 10/22 females had FSH >40 IU/mL x2 - 1 female had spontaneous pregnancy and miscarriage - 1 pregnancy from embryo cryopreservation -74% of males had azoospermia - testosterone levels were all normal - 4/21 males fathered pregnancies; 1 required IVF

ATG, anti-thymocyte globulin; AMH: anti-mullerian hormone; FSH, follicle stimulating hormone; GvHD, graft vs host disease; HSCT, hematopoietic stem cell transplant; HU, hydroxyurea; IVF, in vitro fertilization; LH, luteinizing hormone; POF, primary ovarian failure; POI, primary ovarian insufficiency.

After transplantation, patients are often placed on additional immunosuppressive medications to reduce the risk for GvHD, such as cyclophosphamide or sirolimus (84, 116). Primary ovarian insufficiency is a common consequence of cyclophosphamide (117, 118), although ovarian reserve appears to be less affected if cyclophosphamide is given prior to menarche (119). Sirolimus has also been shown to significantly reduce sperm counts and fertility rates in male organ transplant recipients (120), lead to gonadal dysfunction and secondary infertility (121), and to negatively impact IVF outcomes (122).

In summary, counseling patients and families on future fertility is complicated by the wide range of conditioning regimens available and poor reporting on gonadal function post-transplant. Furthermore, impact on gonadal function may be highly variable even for the same conditioning regimen, with some patients experiencing rapid gonadal failure necessitating hormonal supplementation and others retaining full function. Long term follow-up and a greater understanding of the interplay between gonadotoxic treatment and gonadal function is needed.

## Fertility preservation

Given the risk of gonadal failure after HSCT, fertility preservation before HSCT should be offered (123). Early counseling, no matter the patient age, is vital to provide patients with the optimal opportunity to protect their reproductive potential. Furthermore, fear of toxicity and sterility are important barriers to HSCT acceptance (124, 125). However, significant access to care remains including provider awareness, patient/family preferences, and financial barriers.

### Male fertility preservation

Fertility preservation is an important consideration for men and their provider to discuss prior to HSCT or even initiating hydroxyurea therapy (126). Male fertility preservation in many ways is more straight forward than for females - ejaculate or testicular tissue do not require stimulation cycles and can be collected nearly immediately, but counseling men about the risk of gonadotoxic agents is less frequently discussed with males than female cancer patients (127, 128).

#### Sperm banking

For post pubertal males, sperm banking can readily be performed *via* ejaculation which allows sperm to be frozen until the patient desires fertility, at which time *in vitro* fertilization could be pursued (129). Given the impact of hydroxyurea on spermatogenesis, it is recommended that hydroxyurea be discontinued several months prior to sperm collection (123). This may allow for normalization of sperm parameters. However, discontinuing hydroxyurea for several months may be challenging for patients with severe SCD. While the process of sperm cryopreservation is relatively simple, informing patients of the risk of gonadotoxic agents is not always discussed or pursued (130). Studies have found low rates of fertility preservation counseling prior to gonadotoxic chemotherapy for cancer (128). However, these findings may not be applicable to adolescent males facing gonadotoxic treatment for their chronic lifelong disease and more research is needed about fertility preservation counseling in this population.

Discussion of fertility preservation and increasing state-mandated coverage of fertility preservation has resulted in an increase in rates of fertility preservation (128). In recent years, several online based companies have emerged that allow for in-home collection for sperm banking wherein the individual receives a kit, ejaculates in their own home into the provided container, and then ships the kit back for cryopreservation (131). With increasing insurance coverage of fertility preservation, awareness of fertility preservation options amongst providers, and online-based sperm preservation companies, hopefully the rates of sperm banking prior to gonadotoxic agents will increase.

#### Testicular tissue preservation

Fertility preservation is more complex if the male has not yet gone through puberty. Indeed, rates of azoospermia are quite high in boys 13 years or younger (132). In these cases, surgery is required to harvest testicular tissue for cryopreservation (133). Only a small amount of testicular tissue is collected, and the procedure is well tolerated with minimal side effects (134). It is important however to note that there is no current ability to use this sperm for future fertility attempts; thus prepubescent testicular preservation is only offered in certain academic centers as part of a research protocol (135). Research into maturation of the testicular tissue and methods of reimplantation are ongoing. The field recently took a major step forward with the successful transplant of frozen rat spermatogonial stem cells into recipient mice, which produced differentiating germ cell types with production of spermatids (136). While this murine study is certainly promising for the future of reimplantation of testicular tissue, parents of boys undergoing gonadotoxic treatments should be aware that much research is still required and previously harvested testicular tissue may not be ready for spermatogenesis at the time when fertility may be desired. Currently, testicular tissue preservation is recommended for patients at significant risk for infertility, including patients with SCD and BTM (137). Operative considerations are discussed below.

### Female fertility preservation

The most important factors determining mode of fertility preservation in a female patient are whether she has undergone menarche and the urgency with which the gonadotoxic treatment is needed. Fertility preservation prior to HSCT for hemoglobinopathies is not usually urgent. This will allow for improved coordination and health optimization prior. In some cases, this may allow time for menarche to occur and thus permit the use of controlled ovarian hyperstimulation and oocyte cryopreservation. For patients who have not yet undergone menarche and for whom waiting until after menarche for transplantation is not feasible due to patient age and disease severity, ovarian tissue cryopreservation (OTC) is the only current option for fertility preservation. For females who have undergone menarche, OTC and oocyte cryopreservation are available. While embryo cryopreservation is another option, it is less likely in the pediatric and young adult population as it requires a sperm source and has greater ethical implications.

#### Oocyte cryopreservation

Controlled ovarian hyperstimulation (COH) requires ten to fourteen days of intensive monitoring and medication administration. Follicular growth is monitored *via* transvaginal or transabdominal ultrasound, and hormone levels are monitored through frequent blood work. Oocytes are then harvested *via* a minimally invasive approach under anesthesia.

Common complications of COH include headache, nausea, abdominal distention, and discomfort. Less commonly, ovarian hyperstimulation syndrome (OHSS) occurs, which may produce venous thromboembolism (VTE), ascites, and cardiopulmonary effusions (138). These consequences of COH are of even greater concern for patients with hemoglobinopathies, who may have altered pain perception and be less able to tolerate the discomfort of COH. Ovarian hyperstimulation syndrome also creates a greater risk to patients with underlying vascular, pulmonary, and renal injury, as they may be less able to tolerate fluids shifts (139). Indeed, COH increases the risk of VOC and ACS in patients with SCD. To date, there are 4 reported cases of acute pain crises during COH (**Table 3**).

**Table 3:** Clinical reports of ovarian tissue cryopreservation for sickle cell disease and beta thalassemia.

Author, Year	Cases	Age	Diagnosis/Genotype^1^	Indication for FPT	OTC	Post HSCT	Need for hormonal supplementation	Outcomes
Donnez et al., 2006 (140)	1	21 years	HbSS	Prior to HSCT (busulfan/ cyclophosphamide)	LSCO	Amenorrheic; FSH 48.2 mIU/mL; LH 18.5 mIU/ml; Estradil <10 pg/ml	Required	- After cessation of HRT, bimonthly FSH, LH, 17beta-estradiol demonstrated anovulation -Part of cryopreserved ovary reimplanted - Resumption of ovarian function, follicular development, menstruation
Roux et al., 2010 (141)	1	20 years	HbSS	Prior to HSCT (busulfan/cyclophosphamide)	LSCO	Clinical and biological POI	Required	Desired pregnancy; had transplant - 4 months after OTT, follicle development - 19 weeks after OTT, stopped HRT and normalized AMH, FSH -spontaneous pregnancy, uncomplicated
Revel et al., 2011 (142)	1	19 years	BTM	Prior to HSCT	LSCO	Clinical and biological POI	Required	- Desired pregnancy; 5 mature oocytes were thawed but did not mature - After 1^st^ OTT, FSH decreased, estradiol increased - After 3 cycles of failed IVF, ovarian tissue stopped responding to induction - Underwent 2 additional OTT - after 14^th^ cycle of IVF, conceived, delivered full term
Revelli et al., 2013 (143)	1	21 years	Transfusion-dependent BT	Prior HSCT (busulfan/cyclophosphamide)	Ovarian cortex harvest	Clinical and biological POI	Required	- Desired pregnancy; discontinued HRT, (FSH 72.3 IU/L, LH 32.1 IU/L) with a drop of E2 levels (12 pg/mL) - 3 months after OTT, E2 79 pg/mL, and FSH levels decreased 46.1 IU/L - Spontaneous pregnancy, full term cesarean delivery
Demeestere et al., 2015 (144)	1	13 years (post pubertal, pre-menarchal)	SCD	Prior to HSCT (busulfan/cyclophosphamide/ATG)	LSCO	POI	Required hormonal supplementation for menarche	- Desired pregnancy; discontinued HRT; FSH 59 IU/l, LH 32 IU/l) - 4 months after OTT: hormone levels FSH 5 IU/l; LH 6 U/l; estradiol E2 166 pg/ml) - 5 months after OTT: started regular menstruation - 2 years after OTT: conceived spontaneously; uncomplicated pregnancy *first case of premenarchal OTC resulting in pregnancy
Pecker et al., 2018 (139)	2	25, 27	SCD	Prior to HSCT	LSCO	NR	NR	1 patient experienced pain crisis after laparotomy
Armstrong et al., 2018 (145)	18 (114 total)	Range 2-13 years	10 SCD 8 BT	Unknown/prior to HSCT	NR	NR	NR	Not reported
Matthews et al., 2018 (146, 147)	1	9 years	BTM	Prior to HSCT	LSCO	Menarche age 15; sporadic menses - Patient trying to conceive 2 years; AMH <0.5ng/mL; FSH 18-67 IU/L	Started on estradiol/ Norgestrel for uterine development, regulation of menstrual cycle	- HRT discontinued after OTT - FSH and LH ranged from pre to post-menopausal range, at 5 mo AMH approached 2 ng/mL - Underwent IVF cycle (anatagonist), 8 oocytes retrieved from transplanted ovary; uncomplicated pregnancy
Poirot et al., 2019 (148)	71 (418 total)	Range 0.3-15 years across all categories	“hemo-globinopathies” - not characterized	NR	NR	NR	NR	-oocytes isolated from the tissue were cryopreserved in 50 cases
Mamsen et al., 2021 (37)	14	Range 2.8-17.4 years	10 BT 4 SCD	NR	LSCO	Two patients underwent OTT-menopausal at time of transplant	NR	- follicle density, morphology, and expression of follicle- and oocyte specific proteins were comparable to an age-matched reference group - 3-4 months after OTT, serum hormone levels normal - 1 patient conceived with IVF, gave birth full term *first case of pre-pubertal follicle resulting in pregnancy
Dolmans et al., 2021 (149)	9 (285 total)	Range 9-44 years across all categories	“hemo-globinopathies” - not characterized	NR	NR	NR	NR	- All patients had OTT -40% spontaneous conception rate
Kristensen et al., 2021 (150)	1,186 total	Range 4mo-44 years across all categories	55 benign hematological disease	NR	NR	NR	NR	- 117 returned for OTT - Patients with benign hematological diseases had highest (35%) return rate
Hanfling et al., 2021 (151)	2	2, 18 years	1 SCD, 1 BT	Prior to HSCT (both on hydroxyurea)	LSCO	NR	NR	- Mature oocytes found at time of OTC
Boga et al., 2022 (114)	1	Not reported						

1- Diagnosis and genotype are those reported in manuscript

OTC, ovarian tissue cryopreservation; FPT, fertility preservation treatment; HSCT, hematopoietic stem cell transplant; OTT, ovarian tissue transplantation; SCD, sickle cell disease; BTM, beta thalassemia major; BT, beta thalassemia; POI primary ovarian insufficiency; NR, not reported; LSCO laparoscopic oophorectomy; ATG, anti-thymocyte globulin; E2, estradiol; HRT, hormone replacement therapy.

Results of COH in patients with SCD are variable, with the number of oocytes retrieved ranging from 4 - 31. Fifteen is considered the minimum number of oocytes to harvest to optimize the change of pregnancy in one cycle (152). However, only 25% of the reported patients reached this goal. No patients in these cohorts underwent multiple cycles for fertility preservation, perhaps reflecting time and monetary constraints (139). There is also a scarcity of published data on ovarian stimulation protocols and outcomes in adolescent patients with and without hemoglobinopathies (153). It is generally recommended to use adult dosing regimens as a guide, adjusting for age, FSH level, and AFC. However, this may require frequent and significant dose adjustments. For example, in their cohort of eight teenage girls, Lavery et al., reported that dose adjustments were needed in 80% of cases (154).

To date, there have been several reports of successful and uncomplicated ovulation induction and IVF cycles for untransplanted patients with BTM. However, there have been no published COH protocols for BTM prior to fertility preservation (155, 156). These authors recommended discontinuing iron chelators prior to ovulation induction (156) as they are contraindicated in pregnancy, but this is not necessary for the purpose of fertility preservation.

#### Ovarian tissue cryopreservation

Ovarian tissue cryopreservation (OTC) is an increasingly utilized method of FPT. As of 2019, OTC is no longer considered experimental by the American Society for Reproductive Medicine (ASRM), although it may be in other countries. As of 2017, there had been 130 live births from OTC (157), with estimates of greater than 200 births as of 2020 (158). Given the younger ages at which HSCT is recommended for patients with hemoglobinopathies, OTC may be the only mode of FPT available.

Ovarian tissue cryopreservation is commonly performed *via* an outpatient laparoscopic surgery in which an ovary, or portion of the ovary is removed. This tissue is then stored until future use at which point ovarian tissue transplantation (OTT) may occur. Since OTC enables preservation of a larger cohort of primordial follicles, ovarian endocrine function may be restored after OTT. Indeed, in a metanalysis of 309 cases of OTT, endocrine restoration, as defined by cyclic menstrual cycles, ovarian follicle growth on ultrasound, or pregnancy, was achieved in 64% of cases. Clinical pregnancy rate after OTT was 57.5% (159).

Importantly, ovarian endocrine function appears to be restored in the small number of reported post-OTT patients with hemoglobinopathies (**Table 4**). In 2006, Donnez et al. was the first to report restoration of ovarian function after orthotopic transplantation in a patient with HbSS. The patient underwent OTC prior to HSCT at age 21 years old. She required hormone supplementation after transplant and eventually had an OTT after which she had resumption of ovarian function, evidenced of follicular development, and regular menstruation (140). These findings have been replicated in several other reports of adolescent patients with hemoglobinopathies, including patients who were prepubertal (37).

**Table 4:** Cases of controlled ovarian hyperstimulation in patients with hemoglobinopathies.

Author, Year	Age	Diagnosis	AFC	Days stimulated	Peak estradiol	Total Gonadotropin IU/d	Trigger	#oocytes retrieved	#oocytes cryopreserved
Dovey et al., 2012 (160)	19 years	SCD	20	6	859	900	Leuprolide 20IU BID	9	8
Lavery et al., 2016 (154)	14 years	SCD	13	14	NR	2625	rHCG	7	7
	15 years	SCD	6	10	NR	1875	rHCG	5	4
	16 years	SCD	18	11	NR	131.5	rHCG	21	16
	16 years	SCD	16	10	NR	1462.5	rHCG	29	25
	16 years	SCD	16	10	NR	1500	rHCG	14	11
	17 years	SCD	20	11	NR	3350	rHCG	5	3
	18 years	SCD	20	10	NR	1875	rHCG	31	30
	18 years	SCD	12	12	NR	3075	rHCG	7	1
Matthews and Pollack, 2017 (146)	23 years	SCD	28	5	1,669	1,125	Leuprolide 80IU 2 doses at 36 and 24 hr	9	8
Pecker et al., 2018 (139)	26 years	SCD	2	13	3567	5850	Leuprolide	21	21
	28 years	SCD	Small follicles	10	244	3000	hCG	11	7 embryos
	32 years	SCD	10	10	983	1875	hCG	14	14
	28 years	SCD	Small follicles	12	815	3300	Leuprolide	4	3
	15 years	SCD	14	13	457	2925	hCG	14 (transabdominal)	12
Boga et al., 2022 (114)	1 patient had embryos cryopreserved, 3 had oocytes cryopreserved								

AFC, antral follicle count; SCD, sickle cell disease; NR, not reported; BID, twice daily.

Spontaneous pregnancy may occur after OTC, although IVF is frequently required. In 2015, Demeestere et al., reported the first live birth from ovarian tissue cryopreserved from a pubertal female who was pre-menarchal. The OTC occurred at age 13. The patient, who had SCD, was confirmed to have primary ovarian insufficiency when she desired to conceive. Two years after ovarian transplantation, the patient spontaneously conceived (144). In 2021, Mamsen et al., reported the first case of pregnancy from OTC performed prior to puberty. The patient who had beta thalassemia underwent OTC prior to HSCT at 9 years old. She returned at 23 for OTT after which she had resumption of ovarian function and was able to conceive with IVF. In a separate case report, a patient required 14 cycles of IVF and 3 separate OTT to achieve a life birth (142). Unfortunately, most studies do not report on infertility rates among patients who have undergone OTT, nor do they report sufficient information on the indication for IVF or the number of cycles to draw conclusions about the chance of spontaneous conception after OTT. Furthermore, patients’ response to HSCT is variable; some patients appear to be completely cured after treatment while others have a less robust response. Individuals who are not cured likely differ in pregnancy outcomes given the higher rate of stillbirth and fetal growth restriction in untreated patients with SCD (161).

OTC has dramatically altered opportunities for fertility preservation in pediatric patients, especially those who are prepubertal. Despite the promise of OTC, outcomes should be viewed cautiously. Globally, few pregnancies have occurred for patients who had OTC prior to puberty. While spontaneous pregnancies occur, they should not be viewed as expected. Furthermore, ovarian tissue grafts have a finite life. It is estimated that ovarian grafts last approximately 2.25 years on average (159). Therefore, periodic OTT may be required throughout a female’s reproductive life.

### Other fertility considerations

Women with SCD and beta thalassemia are at increased risks for obstetric complications including maternal mortality, intrauterine fetal demise, preeclampsia, preterm delivery, and spontaneous miscarriage (162–167). These risks are partly due to high rate of comorbidities associated with these hemoglobinopathies, i.e., hypercoagulability. For women with significant comorbidities who wish to have biologic children, the option for surrogacy should be discussed along with appropriate preconception counseling with maternal-fetal medicine specialists.

Another important component of fertility treatment is the discussion of genetic testing. Individuals who carry mutations for hemoglobinopathies should be offered preimplantation genetic testing (PGT) to reduce the risk of an affected offspring. While patients may not be ready for parenthood soon after their fertility preservation, education on surrogacy and PGT may be helpful in informing patients and their families on the full scope of fertility options.

## Discussion

### Preoperative and post-operative risk management

Both surgery and COH contribute to fluid shifts and hypercoagulability, which increase the risks for adverse outcomes among patients with SCD. It is estimated that 5% of pediatric patients experience postoperative VOC and ACS (168, 169), and moderate to severe OHSS occur in 1-5% of all COH cycles (138). To date, there have been six adverse outcomes reports from FPT: (**Table 5**): one episode of mild OHSS, four episodes of acute pain crises, and one episode of ACS requiring intubation and intensive care unit admission. All adverse events occurred in patients with SCD, and the more severe adverse events occurred in older patients with more comorbidities. Patients with BTM who have undergone a splenectomy are at increased risk of post-operative infections and those with hemosiderosis induced heart failure are more prone to fluid overload. However, these risks are less well described than for SCD. To date, no adverse events have been reported in pediatric SCD FPT or in any BTM patient undergoing FPT.

**Table 5:** Adverse outcomes associated with fertility preservation treatment in patients with sickle cell disease.

Authors	Age	Type of FPT	Complication	Management
Dovey et al., 2012 (160)	19	COH	Acute pain crisis starting immediately post oocyte retrieval	Hospital admission
Lavery et al., 2016 (154)	18	COH	Mild OHSS 4 days post retrieval	Supportive care
Matthew and Pollack, 2017 (146)	23	COH	Acute pain crisis on day 6 of COH	Exchange transfusion; cycle continuation
Pecker et al., 2018 (139)	26	COH	ACS, respiratory failure, bacteremia	Intubation, intensive care unit admission, pain control, antibiotics
	27	OTC	Acute pain crisis	Red Cell exchange
	28	COH	Acute pain crisis on day 6 of COH	Hospital admission; IV hydration; pain control

FPT,-fertility preservation treatment; COH,- controlled ovarian hyperstimulation; OTC,-ovarian tissue cryopreservation; OHSS,-ovarian hyperstimulation syndrome.

Preoperative and postoperative optimization are vital to reduce procedural complications and to reduce the risk of cancellation of high stakes cycles. While there are no standardized protocols for preoperative management, there are general principles which should be followed. Below, we discuss the available literature and include a protocol created by our center for management of COH in patients with SCD (**Table 6**).

**Table 6:** Perioperative considerations.

Preoperative	Perioperative	Post-operative
- Coordination with anesthesia and hematology - Develop pain management plan in coordination with hematology - Consider exchange transfusion - Consider HU discontinuation - IF HU naïve and planning on starting HU prior to HSCT, perform FP prior - Consider prophylactic anticoagulation if history of VTE	- First or early start case - Clear liquids up to 2 hours prior to start time - IV hydration when fasting and prior to anesthesia - Minimizing hypothermia in pre-operative space, in the operating room, and in recovery - Avoidance of dexamethasone for nausea	- Early pain management - Early incentive spirometry - Discontinue IV hydration when tolerating by mouth

HU, hydroxyurea; HSCT, hematopoietic stem cell transplant; FP, fertility preservation VTE, venous thromboembolism; IV, intravenous

#### Preoperative/preprocedural planning

Prior to FPT, coordination with the patient’s hematologist is vital for procedural optimization and postprodcedural management. Universal preoperative anesthesia consult is not warranted (170) but should be considered based on patient’s medical comorbidities. Ensuring that children are up to date on disease-specific screening (i.e. transcranial Doppler ultrasound) is also recommended, although this is expected if the patient is in the process of undergoing HSCT. Patients may have a history of prior VTE, and thus an anticoagulation plan must be considered when planning for both surgery and COH. To reduce overall surgical risks, coordinating with other procedures, such as port placement, should be considered.

Creating a COH stimulation protocol that optimizes oocyte yield while minimizing risk of OHSS is vital. In our practice, we use either hCG or a gonadotropin-releasing hormone agonist for trigger to reduce the risk of OHSS (139). Given the unclear impact of hydroxyurea on ovarian reserve, oocyte quality and embryo development (171), we also recommend discussing medication discontinuation prior to FPT.

Data on the benefits of preoperative transfusion in patients with SCD is conflicting (172–176). Nevertheless, most experts recommend transfusion for a hemoglobin level ≥9-10 g/dL and exchange transfusions for hemoglobin S <30% (170, 177), and these are the benchmarks that our group has recommended for management of COH (139). For patients who receive regular transfusions or exchanges, these should be continued in the immediate preoperative period. If transfusion or exchange is planned, coordination with blood bank specialists who are familiar with the patient’s transfusion history and have knowledge of any red blood cell autoimmunization or alloimmunization is recommended (139). Decisions on if and when transfusions or exchanges are recommended should be discussed with the patient’s hematologist.

#### Perioperative

Triggers for sickling should be minimized in the perioperative setting, including dehydration, acidosis, hypoxia, and hypothermia. To prevent dehydration, it is recommended that patients avoid prolonged fasting, consume clear liquids up to two hours prior to their procedure, and receive IV hydration while fasting. Our group recommends administering IV hydration prior to anesthesia administration (139). Normothermia through use of body temperature monitoring systems, blankets, and ambient temperature control is recommended. Monitoring oxygen saturation is paramount, and supplemental oxygen used when indicated. Glucocorticoids, such as dexamethasone, should be avoided as they may precipitate pain crises (139, 178, 179).

#### Postoperative

Use of incentive spirometry, chest physiotherapy, and early ambulation in the postoperative period is widely recommended to reduce the risk of ACS (139, 170). Early pain control is another important facet to postoperative management. Patients with chronic pain, such as in SCD, may have altered pain perception and may already be taking daily narcotics. Preoperative discussion with the patient’s hematologist is absolutely critical when determining postoperative pain regimen. Narcotics are often first line agents, although patient-controlled analgesia may be warranted (170). Understanding the patient’s recent pain history may help to predict postoperative complications, as patients with recent hospitalizations for crises are more likely to have postoperative crises (180).

### Health care and research disparities

Hemoglobinopathies are the most commonly inherited monogenetic diseases, yet research and funding do not reflect the prevalence of the diseases (125, 181). For example, sickle cell related variables are not collected in large health outcomes databases and no robust dataset exists for hemoglobinopathies and fertility (28), thereby limiting providers’ ability to offer evidence based care.

No studies to date have evaluated access to FPT care for patients with hemoglobinopathies (125). However, fertility preservation is not commonly utilized prior to HSCT. For example, in a claims database study of over 400 adults who underwent HSCT, only 7% had claims for fertility preservation services before their transplant (182). A significant barrier to care, especially in the adolescent population is timely referrals. Pediatric providers may not be aware of infertility risks and may feel poorly equipped to discuss fertility preservation and uncomfortable discussing reproductive health with patients and their families (183–185), especially with rapidly evolving recommendations and practices.

For patients who do receive a referral for fertility preservation, the cost of FPT may be a significant barrier. The average cost of COH for fertility preservation is over $12,000 (186, 187), and the cost of laparoscopic oophorectomy is comparable (186). Storage fees for cryopreserved oocytes and ovarian tissue, which may be over twenty years, further adds to required costs. Whereas programs such as Livestrong exist to assist patients in fertility preservation prior to cancer treatment, no such national program exists for hemoglobinopathies. As of 2022, 12 states mandate coverage for fertility preservation prior to gonadotoxic treatment (188). However, mandates often do not cover government assistance such as Medicaid and Medicare, and some states such as Utah require that the patient has a cancer diagnosis, thereby disqualifying many patients with SCD or BTM. Some have argued for the need to change institutional programs (125) to provide coverage for patients with SCD. Ultimately, advocating for legislation change both on the local and national level is needed to expand coverage for this population.

Adoption and utilization of oncofertility patient navigators (189) may help to reduce some of these barriers to fertility preservation among patients with hemoglobinopathies. Patient navigators help guide patients and their families through fertility preservation, from identifying an in-network clinic, expediting fertility evaluation, providing education about different fertility options, and referring to different support groups (190). Through advocating for patients and their families, navigators may play a vital role in empowering them to make the right fertility preservation decisions for their circumstances and goals.

## Conclusions

Sickle cell disease and beta thalassemia are the most common and morbid hemoglobinopathies. Disease modifying and curative treatments have improved quality of life and increased the chance of living into adulthood. However, many of these treatments negatively impact fertility and normal pubertal development. Fertility preservation should be discussed with all patients and families considering disease modifying and curative therapies. In very young children in which fertility preservation may be challenging, the risks and benefits to delaying HSCT for greater maturation should be discussed. Counseling patients and families about future fertility must take into consideration the patient’s disease, treatment history, and planned treatment, acknowledging current knowledge gaps. Preparing for fertility preservation must also include a multidisciplinary approach to optimize patient outcomes while reducing surgical and procedural risks. Further research and advocacy are needed to improve patient care and future fertility.

## Author contributions

MC, BB, LP and TK contributed to conception and outline of the manuscript. BB wrote the first draft of the manuscript. TK, LP and MC wrote sections of the manuscript. All authors contributed to manuscript revision, read, and approved the submitted version.

## Conflict of interest

The authors declare that the research was conducted in the absence of any commercial or financial relationships that could be construed as a potential conflict of interest.

## Publisher’s note

All claims expressed in this article are solely those of the authors and do not necessarily represent those of their affiliated organizations, or those of the publisher, the editors and the reviewers. Any product that may be evaluated in this article, or claim that may be made by its manufacturer, is not guaranteed or endorsed by the publisher.
